# Screening and Identification of putative long non coding RNAs from transcriptome data of a high yielding blackgram (*Vigna mungo*), Cv. T9

**DOI:** 10.1016/j.dib.2018.01.043

**Published:** 2018-02-20

**Authors:** Pankaj Kumar Singh, Sayak Ganguli, Amita Pal

**Affiliations:** a Division of Plant Biology, Bose Institute, Kolkata 700054, India; b Theoretical and Computational Biology Division, AIIST, Palta 743122, India

**Keywords:** Blackgram, Long non-coding RNA, Legumes, RNA sequencing data

## Abstract

Blackgram (*Vigna mungo*) is one of primary legumes cultivated throughout India, Cv.T9 being one of its common high yielding cultivar. This article reports RNA sequencing data and a pipeline for prediction of novel long non-coding RNAs from the sequenced data. The raw data generated during sequencing are available at Sequence Read Archive (SRA) of NCBI with accession number- SRX1558530

**Specifications Table**

**Table:** 

Subject area	*Biology*
More specific subject area	*Plant molecular biology*
Type of data	*Sequence Data*
How data was acquired	*High throughput sequencing*
Data format	*Raw reads*
Experimental factors	*High Yield Cultivar*
Experimental features	*RNA sequencing from total isolated RNA, followed by computational prediction of long non-coding RNA*
Data source location	*Kolkata, West Bengal, India*
Data accessibility	https://www.ncbi.nlm.nih.gov/sra/SRX1558530

**Value of the data**

• This is the first report of long non-coding RNAs in *Vigna mungo.*
• This study will enable researchers to identify lncRNAs of interest in a high protein yielding legume, *Vigna mungo*.
• This article also contains a pipeline for identification of long non-coding RNAs in *Vigna mungo* an in depth analysis with some adjustments which may pave the way for identification of lncRNAs in other non model plants as well.

## 1 Data

This works reports the long non-coding RNAs identified in common Indian cultivar of *Vigna mungo* (Blackgram) Cv. T9. This cultivar is widely cultivated in different states of India due to high agronomic yield; however, it is highly susceptible to Mungbean Yellow Mosaic India Virus (MYMIV) infection mediated by the vector whitefly (*Bemisia tabaci).*

## 2 Experimental design, materials and methods

### 2.1 RNA isolation and RNA sequencing

Sample preparation for RNA isolation was done as described by Kundu et al. [1]. Total RNA was extracted from prepared sample using Trizol reagent (Invitrogen, Carlsbad, CA) following the manufacturer's instruction, followed by DNase-I treatment (Sigma-Aldrich, USA) and purification using a RNeasy Plant Mini Kit (Qiagen, USA). Qualitative and quantitative assessments of the extracted Total RNA were performed using Agilent 2100 Bioanalyzer (RNA Nano Chip, Agilent). RNA samples were transferred to Genotypic Technologies Pvt. Ltd. (Bangalore, India) for transcript library preparation and for performing high throughput sequencing using Illumina NextSeq. 500 platform. Data generated during this experiment was submitted to Sequence Read Archive (SRA) of National Centre for Biotechnology Information (NCBI) under accession no SRX1558530.

### 2.2 Bioinformatics analysis and long non-coding RNA prediction

**Fig. 1:**
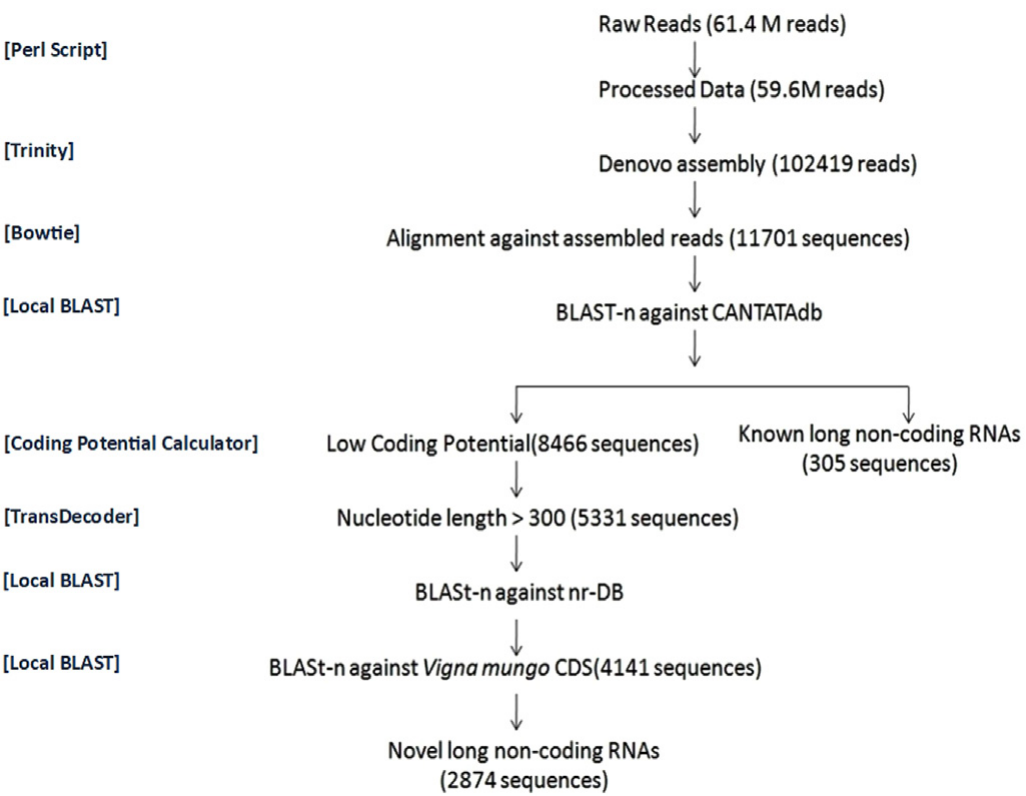
A flowchart representing novel lncRNA prediction used for this dataset.

**Fig. 2:**
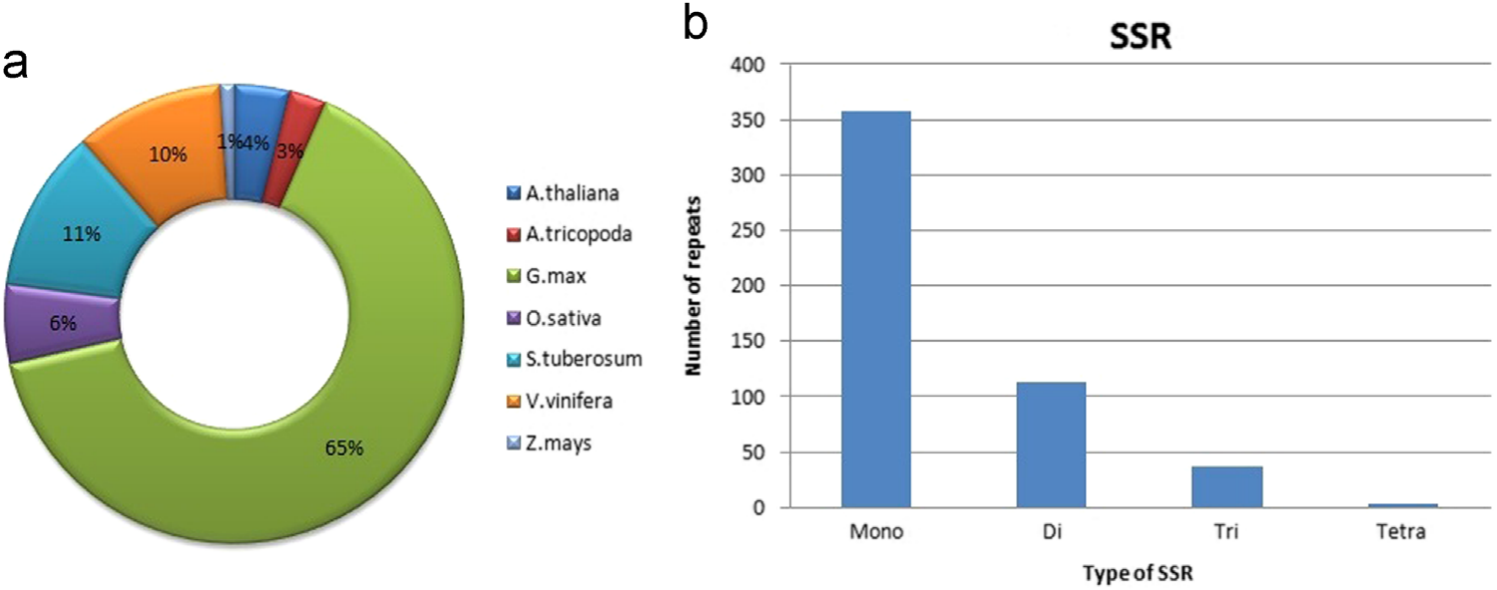
(a) Doughnut representing similarity of known lncRNAs with lncRNA of different plants from CANTATAdb, (b) represents a histogram of number of SSRs predicted for mono,di,tri,and tetra nucleotide repeats. Majority of SSRs are mono nucleotide repeats.

The pipeline shown in Fig. 1 was followed to identify the long non coding RNAs.First raw reads were processed for removal of low quality reads using in house Perl scripts, followed by *de-novo* assembly of transcripts using Trinity [2].*De novo* transcript statistics are provided in Table 1. Processed reads were aligned against assembled transcripts using Bowtie2 [3]. Further BLAST-n [4] was performed against CANTATAdb [5]. Annotated (305 RNAs, Supplementary file 1) and unannotated transcripts (8455 RNAs) were separated. Highest similarities were found with *Glycine max* (65%) (Fig. 2A). Unannotated transcripts were analyzed further, coding potential of transcripts was calculated using CPC Calculator tool [6] and transcripts having low coding potential were selected. Transcripts having length of over 300 bps were selected as suitable candidates for further analyses using TransDecoder. The retained transcripts were again subjected to BLAST nr-Db to establish their non coding character; reads were further searched for similarity against *Vigna mungo* cds (generated via transcriptome sequencing; results unpublished). Remaining 2874 (Supplementary file 2) reads are being proposed as potential novel long non-coding RNAs. This entire pipeline for novel lncRNA prediction is illustrated in Fig. 1.

**Table 1:** Assembly statistics for *De novo* assembly.

**Transcriptome assembly:**	**Statistics**
Transcripts generated:	102,419
Maximum transcript length:	19,931
Minimum transcript length:	201
Average transcript length	1115.17
Total transcriptss length:	114,214,721 (114.2 MB)
Total number of non-ATGC characters:	0
Percentage of non-ATGC characters:	0
Transcripts > 500 b:	58,588
Transcripts > 1 Kb:	38,336
Transcripts > 10 Kb:	44
n50 value:	1976
n90 value:	436
Number of reads used:	29,522,404
Total number of reads:	29,799,540
Percentage of reads used:	99.07

#### 2.2.1 Prediction of SSR markers in novel lncRNAs

Simple sequence repeats were predicted using MISA-MIcroSAtellite identification tool [7]. Ten repeating units for mono nucleotide, 6 repeating units for di nucleotide and 5 repeating units for tri-, tetra-, penta- and hexa nucleotide were chosen as parameters for mining the SSR markers. Details of mined SSRs has been provided in Fig. 2B.
