# Supplementary material for: Screening and Identification of putative long non coding RNAs from transcriptome data of a high yielding blackgram (Vigna mungo), Cv. T9
Source: Data Brief. 2018 Feb 20;17:459–62. doi: 10.1016/j.dib.2018.01.043 (PMC5988335; doi:10.1016/j.dib.2018.01.043)
Supplement: Supplementary file 1 — Supplementary material [file mmc1.zip › Conflict of Interest.pdf]

Conflict of Interest : None
